# Death by Chloroform and Alleged Death by Ether

**Published:** 1872-12

**Authors:** Henry J. Bigelow

**Affiliations:** Boston


### DEATH BY CHLOROFORM, AND ALLEGED DEATH BY ETHER.

BY HENRY J. BIGELOW, M.D., Boston.

In the great rarity of " death from ether," the case reported iu the *Medical Record* of October 1st, will probably be quoted, and perhaps also confounded with the usual "death from chloroform," which is quite different. For this reason, we believe that the reporter of this case will pardon the following remarks, based in part upon the omissions of his narration.

Nobody doubts that a patient can be narcotized with ether until life is extinguished; and that this result will follow with a rapidity proportioned to his want of health and strength.

Hence, in administering an anæsthetic to a patient reduced, for example, by delirium tremens, by long disease of the bladder, of a joint, or by fracture of the hip, care should be exercised. Here a sudden syncope, or even a more gradual falling off, whether of pulse or respiration, may mean something. In the course of ten or fifteen minutes, the vital force, after having given fair warning, may yield with unexpected and startling suddenness.

Exhausting and protracted influences like these are far more potent in their depressing effects upon the sytem, especially in diminishing its power of supporting anæsthesia, than a recent injury to a more robust subject. Such a patient may be cold, pulseless, and nearly insensible, as a result of one or more limbs crushed by railroad accident some hours before, but is usually stimulated by ether inhalation. As a rule his pulse comes up, and he undergoes his amputations well.

If the narcotism* of ether is ever fatal to an aged or exhausted subject, in the way described, it should be remembered that chloroform would be more fatal to the same patient under the same circumstances.

*Death from narcotism,* although it may be heralded as "death from ether," is not death from any property peculiar to ether. It is death from a depressing influence, common to ether, chloroform, alcohol and opium, and is most likely to occur with the strongest of these agents. Moreover, *precaution will prevent it.*

But "*death from chloroform,*" which has given to chloroform its doubtful reputation, is a different thing, which *no human forethought can avert.* It is death from a small and usual dose of this powerful agent; death to a healthy person, sometimes as sudden as by a stroke of lightning, without warning of any sort—*by shock.* We hold that *ether has never produced such a result,* but always gives fair and adequate notice of the approach of danger.

If these views are correct, it follows that a certain small proportion of "deaths from chloroform," so-called, should be subtracted from its bill of mortality, and credited to a "narcotism" common to ether and chloroform. But a similar analysis will wholly expunge the two or three fatal cases, for which ether has been by some persons held to answer. Chloroform will still be reponsible for its monthly record of death by "*shock,*" small in its percentage it may be, but inevitable, while ether will then have a clean bill of health.

Let us now, with a desire to give ether a candid hearing, and also because it, in some measure, illustrates so-called "death from ether," consider the fatal case which occurred in the hands of the house-surgeon and his assistant at the Bellevue Hospital.

---

*The term "narcotism" expresses well enough for present purposes, the gradual effect upon the system of anæsthesia in general, while "shock" may stand for the sudden or toxic effect of chloroform.

A man of 68, with a fracture just below the trochanter, of eighteen days standing, having the lower lobe of his right lung œdematous, and its " lower portion in a state of red hepatization," there being, also, " emphysema," and thickening of the large bronchi," had ether " slowly and carefully " administered, during " perhaps ten minutes," for the application of a plaster bandage. The patient was then " fully under its influence."

"A few turns of the plaster had been made, when the patient's breathing was observed to be rather frequent and gasping. The thorax was compressed two or three times, and the patient's breathing again became normal. As these symptoms not rarely occur during etherization, they excited no special alarm. The ether was, however, withheld from the patient four or five minutes, his respiration and pulse being normal. As he then began, however, to move about and his muscles were rigid, the ether cone was again applied."

This is not quite clear. The patient was " fully " etherized. Everything, the pulse included, is said to have been as it should be, except the breathing, which became " rather frequent and gasping." Now a patient in his senses, with his chest filled with water, for example, may breathe in this way. But is a patient likely to breathe of a sudden more rapidly when he is fully narcotized? We do not agree with these symptoms " not rarely occurring during etherization." With the experience of many thousand cases, we have never seen them. A patient with ether may be faint,* as, perhaps, this one was, or, when fully under its influence, may breathe more slowly, or suspend his respiration, or snore ; or when he does not get air enough, may have spasm of the glottis from asphyxia, producing more asphyxia, discoloration, and general muscular rigidity, but, so far as we know, never an accelerated respiration with gasping, when fully etherized, the pulse being full and regular.

Nevertheless, these, or other appearances not mentioned, led to an attempt at artificial respiration, and to the suspension of the ether inhalation during four or five minutes.

* Symptoms of pulse and respiration, practically those of syncope, are not unfamiliar to persons in the habit of etherizing. The operating chair at the Massachusetts General Hospital, in use since 1854, was furnished with a hinged back and foot-board, to provide against this contingency. The patient can be at once laid flat.

The rest of the story is given in a few words.

"In a minute or two my assistant, who was giving the ether, observed the pupils to be dilating rapidly, and the breathing to cease. His heart was still beating, however."

We naturally ask, how, during this critical and not very brief interval, was the pulse—a most reliable pilot among the possible dangers of etherization. There is no mention of it.

Such signs as those mentioned above are hardly compatible with the continuance of a full and regular pulse, and cannot but feel that the pulse at the wrist, if kept in hand, would have given warning of their approach.

For ourselves, if called upon to indicate, in the absence of complete evidence, the probable cause of this death, we should perhaps reärrange the statement somewhat, as follows:

A man of nearly 70 years, reduced by a fracture near the trochanter, of eighteen days' standing, and by pneumonia, was subjected to a somewhat protracted inhalation before coming under the influence of ether. At the end of ten or twelve minutes, his breathing became so feeble and irregular that etherization was suspended and artificial respiration was resorted to. In the course of four or five minutes more, there being some muscular action, the respiration being also stronger, and the pulse better, the ether was again administered, but the same bad symptoms soon supervened. On examination, the pupils were now found to be dilated. The heart was still beating (although there was probably no pulse at the wrist), but attempts at resuscitation were this time ineffectual.

Such an account would better accord with previous experience. The vitality of a patient enfeebled by age and disease, proves to be easily depressed, and after giving to the operator good and sufficient warning of his enfeebled condition, he succumbs; an occurrence which may serve as a fresh and salutary lesson to the surgeon to exercise care during anæsthesia by ether, and still more by chloroform, of a system thus depressed; but an accident we believe to be impossible to ether, with the pulse held and the respiration attended to.

On the other hand, we repeat, no precaution yet devised by human ingenuity will prevent the insidious shock of chloroform, in even a small dose, from occasionally and abruptly killing a healthy subject. This is the peculiar and usual *death from chlo-*

*roform*, and of its approach, neither pulse nor breathing gives indication.

Ether is so safe when used liberally, and even prodigally, that after a time, the practitioner may perhaps fail to be quite alive to the possible dangers which are inseperable from every remedy of any power. With a tithe of the extreme but vain precaution of English practitioners against the shock of chloroform, we hold that ether would be innocuous. In the above case, an exceptional degree of caution, suggested by the symptoms, might perhaps have saved the patient; but it is important to say that we find nothing in the account to show that the house-surgeon failed to exercise so much care, on the whole, as is common in the administration of ether, and such as usually insures to the patient immunity from accident.—*Boston Medical and Surgical Journal.*

